# Induction of interferon-β and interferon signaling by TRAIL and Smac mimetics via caspase-8 in breast cancer cells

**DOI:** 10.1371/journal.pone.0248175

**Published:** 2021-03-26

**Authors:** Victoria Granqvist, Christian Holmgren, Christer Larsson

**Affiliations:** Lund University, Translational Cancer Research, Medicon Village, Lund, Sweden; Bauer Research Foundation, UNITED STATES

## Abstract

Breast cancer prognosis is frequently good but a substantial number of patients suffer from relapse. The death receptor ligand TRAIL can in combination with Smac mimetics induce apoptosis in some luminal-like ER-positive breast cancer cell lines, such as CAMA-1, but not in MCF-7 cells. Here we show that TRAIL and the Smac mimetic LCL161 induce non-canonical NF-κB and IFN signaling in ER-positive MCF-7 cells and in CAMA-1 breast cancer cells when apoptosis is blocked by caspase inhibition. Levels of p52 are increased and STAT1 gets phosphorylated. STAT1 phosphorylation is induced by TRAIL alone in MCF-7 cells and is independent of non-canonical NF-κB since downregulation of NIK has no effect. The phosphorylation of STAT1 is a rather late event, appearing after 24 hours of TRAIL stimulation. It is preceded by an increase in IFNB1 mRNA levels and can be blocked by siRNA targeting the type I IFN receptor IFNAR1 and by inhibition of Janus kinases by Ruxolitinib. Moreover, downregulation of caspase-8, but not inhibition of caspase activity, blocks TRAIL-mediated STAT1 phosphorylation and induction of IFN-related genes. The data suggest that TRAIL-induced IFNB1 expression in MCF-7 cells is dependent on a non-apoptotic role of caspase-8 and leads to autocrine interferon-β signaling.

## Introduction

Breast cancer can be grouped into different subtypes, where expression of the estrogen receptor (ER) and amplification of human epidermal growth factor receptor 2 (HER2) are important markers for selecting hormonal therapy or therapies targeting HER2 [1]. Based on global mRNA expression, tumors have been classified in intrinsic subtypes: luminal A and B, normal-like, basal, and HER2-enriched [2, 3]. Luminal A, B and normal-like tumors generally express ER [2, 3], with luminal A having the best prognosis [4]. HER2-enriched tumors frequently have amplification of the HER2 gene and basal-like tumors in general lack expression of both ER and progesterone receptors as well as HER2-amplification [2, 3].

Facilitation of apoptosis induction is one way to suppress cancer growth. Smac mimetics, small molecules that mimic functions of second mitochondrial activator of caspases (Smac), have been developed to inhibit certain inhibitor of apoptosis proteins (IAPs), such as cellular IAP (cIAP) 1 and 2, and X-linked IAP (XIAP) [5]. IAPs have a RING domain with E3 ligase activity, which allows them to mediate ubiquitination of themselves and target proteins such as NF-κB-inducing kinase (NIK) [6], receptor interacting proteins kinases (RIPs) [7], and caspases [8]. This allows IAPs to suppress apoptosis and modulate the NF-κB signaling pathways [9–11]. The expression of IAPs has been reported to be elevated in several cancer types. For example, enhanced expression of XIAP is associated with poor prognosis in breast cancer [12]. However, treatment of cancer cells with Smac mimetic as a single therapy has in general not been found to be effective, which has led to it being tested as part of a combination therapy [13].

The death receptor ligand TNF-related apoptosis-inducing ligand (TRAIL) could potentially be used in combination with Smac mimetics since TRAIL preferentially targets cancer cells while sparing normal cells [14]. TRAIL stimulates the extrinsic apoptotic pathway by binding to its receptors DR4 (TRAIL-R1) and DR5 (TRAIL-R2) [15, 16]. This results in the formation of the receptor-bound death-inducing signaling complex (DISC), consisting of Fas-associated death domain (FADD) and caspase-8, which subsequently results in caspase activation and apoptosis [17]. TRAIL may also promote NF-κB signaling [18] which can be both pro-survival [19] and inflammatory, exemplified by induction of pro-inflammatory cytokines and chemokines, such as interleukin (IL) -6, -8, and CXC motif ligand 1 (CXCL1) in a NF-κB- [20], or a FADD- and caspase-8-dependent manner [21, 22]. Here, FADD and caspase-8 form a cytosolic complex, called the FADDosome, where caspase-8 does not have an apoptotic role but rather acts as a scaffold, which may recruit RIP1, to mediate the production of inflammatory cytokines and chemokines. TRAIL signaling has also been shown to induce expression of interferon-β (IFN-β) and IFN-regulated genes [23].

IFN-β is a type I IFN and is generally induced upon viral infection [24]. It binds to its heterodimeric receptor composed of interferon alpha/beta receptor (IFNAR) subunits 1 and 2 [25]. Upon ligation, IFN-β activates the Janus tyrosine kinases, which in turn phosphorylate signal transducer and activator of transcription 1 (STAT1) and STAT2 [26]. Subsequently, STAT1 and STAT2 will dimerize and form a complex with IFN-regulatory factor 9 (IRF-9), called IFN-stimulated gene factor 3 (ISGF3), which will bind to IFN-stimulated response elements (ISREs) and act as a transcription factor of ISGs [27].

IFN signaling has been found to affect cancer aggressiveness. For breast cancer the association seems to differ depending on ER status. In ER-positive breast cancer cells, IFN signaling is associated with resistance to radiotherapy and hormonal therapy [28, 29]. On the other hand, IFN signaling in ER-negative breast cancer is associated with chemotherapy response [30], and longer distant metastasis-free survival during chemotherapy *in vivo* [31]. Here, we show that the combination of Smac mimetics and TRAIL induce IFN-β production in ER-positive MCF-7 breast cancer cells, which do not respond with apoptosis, and in CAMA-1 cells when apoptosis is blocked. The data indicate that this can be followed by autocrine IFN-β stimulation.

## Results

### TRAIL together with Smac mimetics induce IFN and NF-κB signaling in breast cancer cell lines

**Fig 1:**
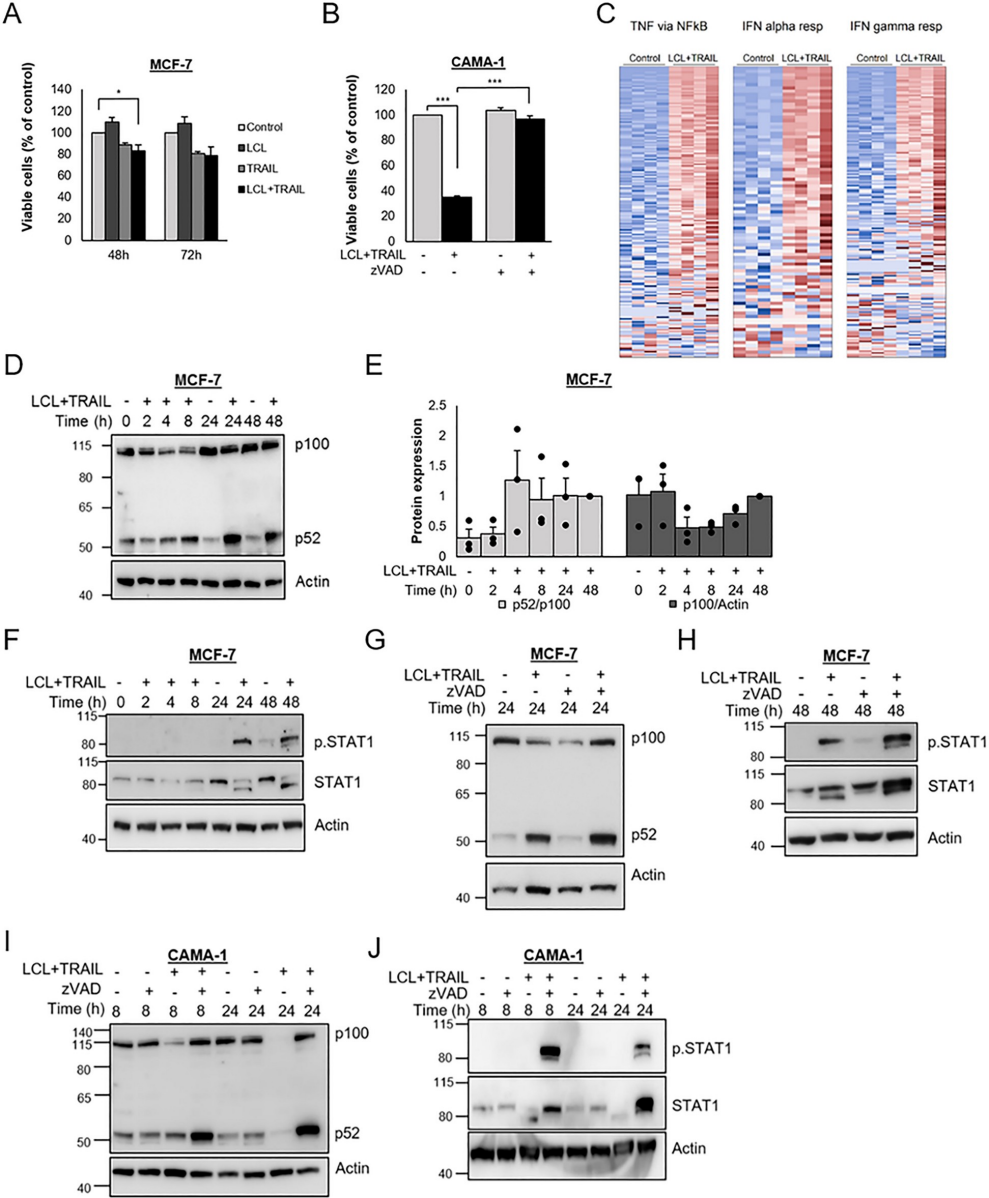
LCL161 and TRAIL induce NF-κB and IFN signaling in breast cancer cells. Cell viability was measured using WST-1 assay for MCF-7 cells treated with LCL161 (10 μM) and/or TRAIL (100 ng/mL) for indicated time points (A) or for CAMA-1 cells treated for 24 h with the same concentrations of LCL161 and TRAIL in the absence or presence of zVAD-FMK (20 μM) (B). Bars represent the percentages of cell viability. Data are mean ± SEM, n = 3. C) RNA was extracted from MCF-7 cells treated with LCL161 and TRAIL for 24 h and was subjected to RNA-seq analysis. The heat map demonstrates the expression in control- and LCL161+TRAIL-treated cells of genes from three Hallmarks (MSigDb) gene sets (TNFA_SIGNALING_VIA_NFKB”, “INTERFERON_ALPHA_RESPONSE”, and “INTERFERON_GAMMA_RESPONSE”). MCF-7 cells were treated with LCL161 and TRAIL (D-H) and pre-treated with zVAD-FMK (20 μM) (G-H) for indicated time points before cells were harvested and protein levels of the NFKB2 gene product p100 and p52 (D, G), or phosphorylated and total STAT1 (F, H) were analyzed by immunoblot. Intensities of bands in D were quantified (E) and normalized to 48 h treatment with LCL161 and TRAIL. Individual data points and mean ± SEM (n = 3) are shown. Protein levels of p100, p52 (I), or phosphorylated and total STAT1 (J) were analyzed in cell lysates of CAMA-1 cells pre-treated with zVAD-FMK followed by LCL161 and TRAIL treatment for indicated time points. * denotes p<0.05 and *** p<0.001 using ANOVA followed by Tukey’s honest significance test.

Treatment of the ER-positive, luminal-like breast cancer cell line MCF-7 with Smac mimetic LCL161 and/or TRAIL for 48 or 72 hours at most had minor effects on cell viability (Fig 1A) which contrasts the ER-positive CAMA-1 cells where the combination induces caspase-dependent cell death (Fig 1B). We therefore took the approach to analyze if the combination induces other effects than cell death in MCF-7 cells. A global mRNA analysis with RNA-seq was done for MCF-7 cells treated for 24 hours with LCL161 and TRAIL. Applying t-test we found that 90 genes were upregulated with a p-value<0.0005 as cut off. These genes were analyzed for enrichment among the Hallmarks sets retrieved from mSigDb [32] using Fisher’s test and a p-value<10^−8^ as cut off. The approach identified three gene sets—“TNFA_SIGNALING_VIA_NFKB”, “INTERFERON_ALPHA_RESPONSE”, and “INTERFERON_GAMMA_RESPONSE” that were enriched for upregulated genes, illustrated by a heat map (Fig 1C), which suggests activation of NF-κB and IFN signaling pathways.

We next more directly analyzed these pathways. Treatment of MCF-7 cells with LCL161 and TRAIL resulted in conversion of p100 to p52 protein after four-eight hours of treatment as indicated by increases in p52 and decreases in p100 levels (Fig 1D). This was followed by increases in p100 and maintained p52 levels suggesting elevated p100 synthesis and ongoing conversion to p52. This is conceivably due to increased mRNA expression since log2 NFKB2 mRNA expression increased from 2.61±0.26 to 5.22±0.24 in the RNA-seq data following 24 hours of LCL161 and TRAIL treatment. Thus, there is activation of the non-canonical NF-κB pathway. The combined treatment with LCL161 and TRAIL also resulted in phosphorylation of STAT1 after 24 and 48 hours (Fig 1E), indicating an activation of the IFN signaling pathway. Caspases can be activated both by TRAIL [33] and Smac mimetics [34]. However, the pan-caspase inhibitor zVAD-FMK rather potentiated the increase in p52 levels (Fig 1F) and phosphorylation of, and increase in STAT1 levels (Fig 1G).

CAMA-1 is another luminal-like, ER-positive breast cancer cell line. However, these cells undergo caspase-dependent apoptosis upon treatment with LCL161 and TRAIL (Fig 1B). To estimate increases in p52 and STAT1 phosphorylation by LCL161 and TRAIL, apoptosis was blocked by the pan-caspase inhibitor zVAD-FMK. As seen in MCF-7 cells, p52 protein levels were elevated (Fig 1H), STAT1 total protein levels were increased, and STAT1 was phosphorylated upon treatment of CAMA-1 cells with the combination of LCL161 and TRAIL together with a caspase inhibitor (Fig 1J).

### Phosphorylation of STAT1 is independent of non-canonical NF-κB and mediated via Janus kinase activity

**Fig 2:**
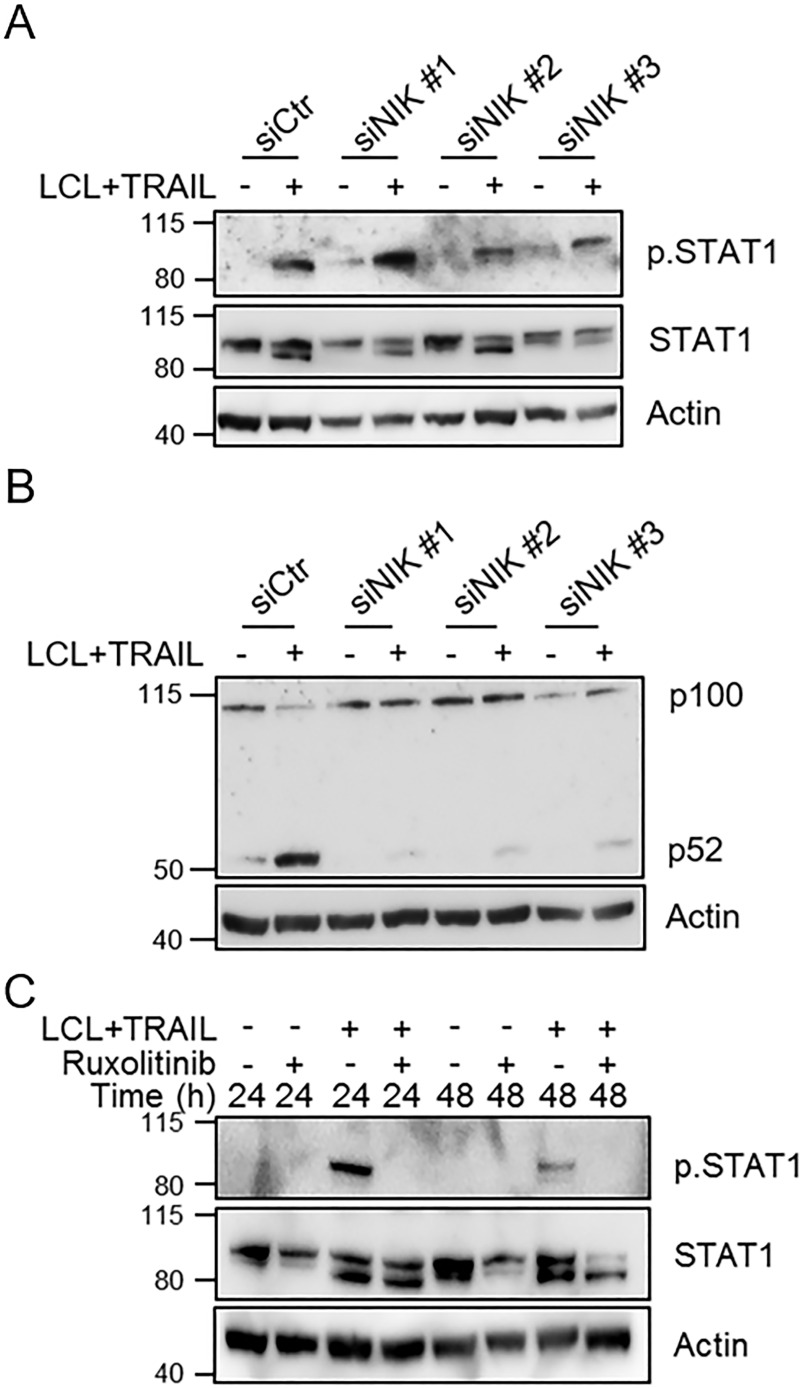
Janus kinase activity, but not non-canonical NF-κB, is involved in LCL161+TRAIL-induced STAT1 phosphorylation. A) MCF-7 breast cancer cells were transfected with three different siRNAs targeting NIK (siNIK #1–3) followed by treatment with LCL161 and TRAIL for 24 h. Phosphorylated and total STAT1 were detected with Western blot. B) Levels of the NFKB2 gene product p52 were analyzed to validate effective knockdown of NIK function. C) The effect of Janus kinase inhibition with Ruxolitinib (20 μM) on STAT1 phosphorylation and increase in STAT1 protein levels was estimated with Western blot. All figures are representatives from three individual experiments.

The increase in p52 protein levels can be detected after eight hours of treatment of MCF-7 cells with LCL161 and TRAIL whereas the phosphorylation of STAT1 requires longer time to become detectable (Fig 1). It is known that NF-κB signaling can induce IFN signaling [35] and we therefore investigated if it participates in causing phosphorylation of STAT1. Downregulation of NIK, the main inducer of the non-canonical NF-κB pathway, did not affect the phosphorylation of STAT1 (Fig 2A). However, it did suppress the induction of p52 indicating that the knockdown, using siRNA, was effective (Fig 2B). We next focused on Janus kinases, which are known to phosphorylate STAT1 upon IFN signaling. Janus kinases were inhibited with Ruxolitinib, which resulted in a total suppression of the STAT1 phosphorylation induced by LCL161 and TRAIL (Fig 2C).

### STAT1 phosphorylation is induced by TRAIL in MCF-7 cells

**Fig 3:**
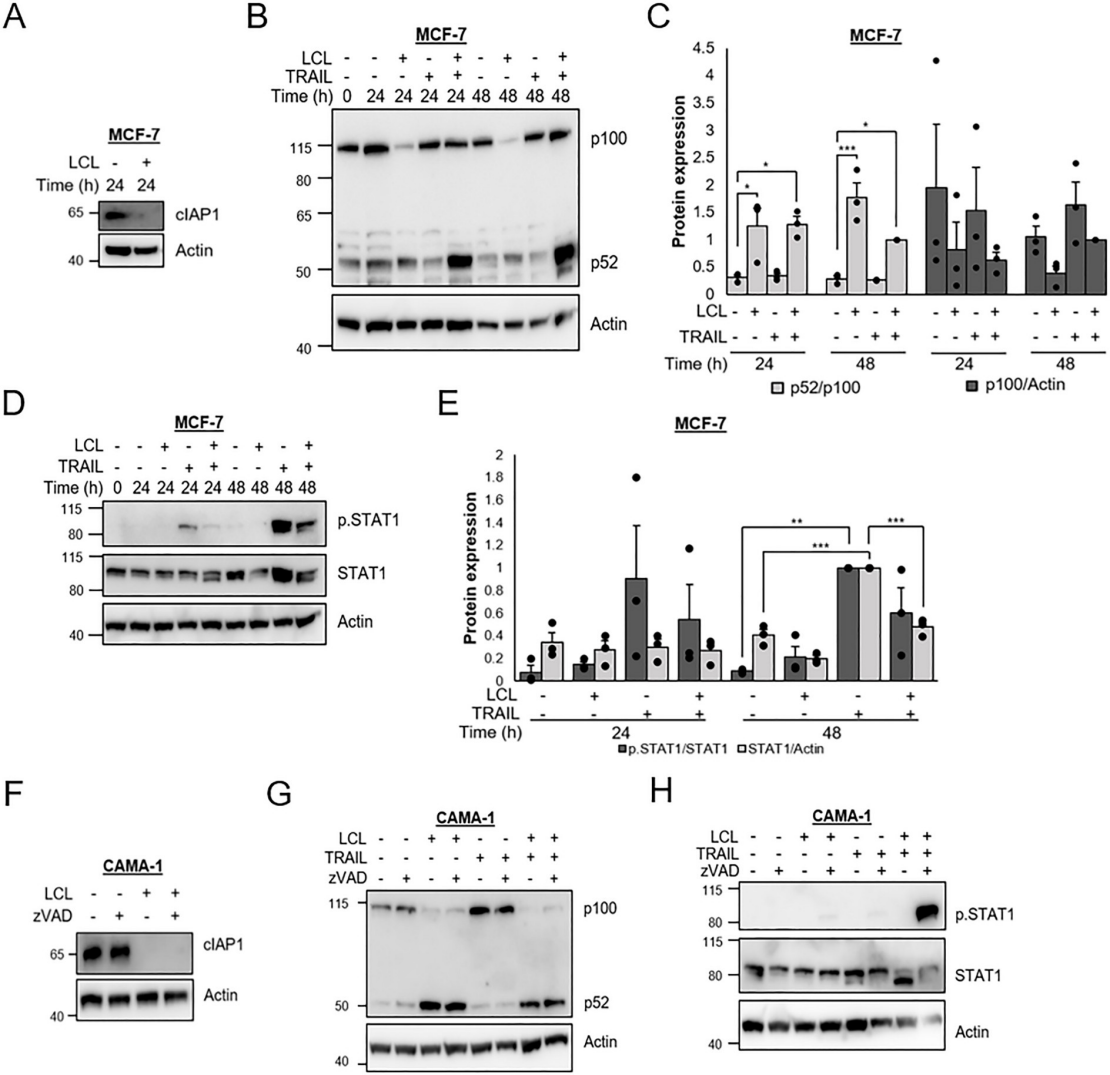
TRAIL is sufficient for induction of STAT1 phosphorylation in MCF-7, but not in CAMA-1 cells. MCF-7 cells were treated with LCL161 (10 μM) and/or TRAIL (100 ng/mL) for indicated time periods and total cell lysates were analyzed by immunoblotting for cIAP1 (A) the NFKB2 gene product p100 and p52 (B) or phosphorylated and total STAT1 (D). Intensity of bands in B and D were quantified (C and E) and normalized to LCL161+TRAIL (C) or TRAIL (E) treatment for 48 h. Protein levels of cIAP1 (F), p100 and p52 (G), or phosphorylated and total STAT1 (H) were measured in CAMA-1 cells that had been treated with LCL161 and/or TRAIL for 8 h in the absence or presence of zVAD-FMK (20 μM). Figures are representative of three independent experiments. Data in C and E indicate individual data points with bars representing the mean and error bars representing SEM, n = 3. * denotes p<0.05, ** p<0.01, *** p<0.001 using ANOVA followed by Tukey’s honest significance test.

Next, we analyzed the individual roles of TRAIL and LCL161 in induction of non-canonical NF-κB and IFN signaling. In MCF-7 breast cancer cells, LCL161 alone induces a slight induction of p52, which is potentiated if it is combined with TRAIL although the p52/p100 ratios are similar for both treatments (Fig 3B and 3C). The effect of TRAIL is conceivably due to an increased p100 synthesis, which was also suggested in Fig 1D, with continued processing to p52.

On the other hand, TRAIL is sufficient for the phosphorylation of STAT1 (Fig 3D and 3E). This is followed by increases in total STAT1 levels, which is evident after 48 hours. In fact, TRAIL alone was more potent than the combination of LCL161 and TRAIL, indicating that LCL161 suppresses the TRAIL effect. The individual roles of LCL161 and TRAIL on non-canonical NF-κB and IFN signaling in CAMA-1 cells were also examined. Here the cells were protected from cell death using zVAD-FMK. We found that LCL161 alone induces an increase in p52 protein levels, which was not markedly affected by simultaneous treatment with TRAIL (Fig 3G). On the other hand, both LCL161 and TRAIL are required for STAT1 phosphorylation (Fig 3H).

### TRAIL induction of IFNB1 may precede STAT1 phosphorylation

**Fig 4:**
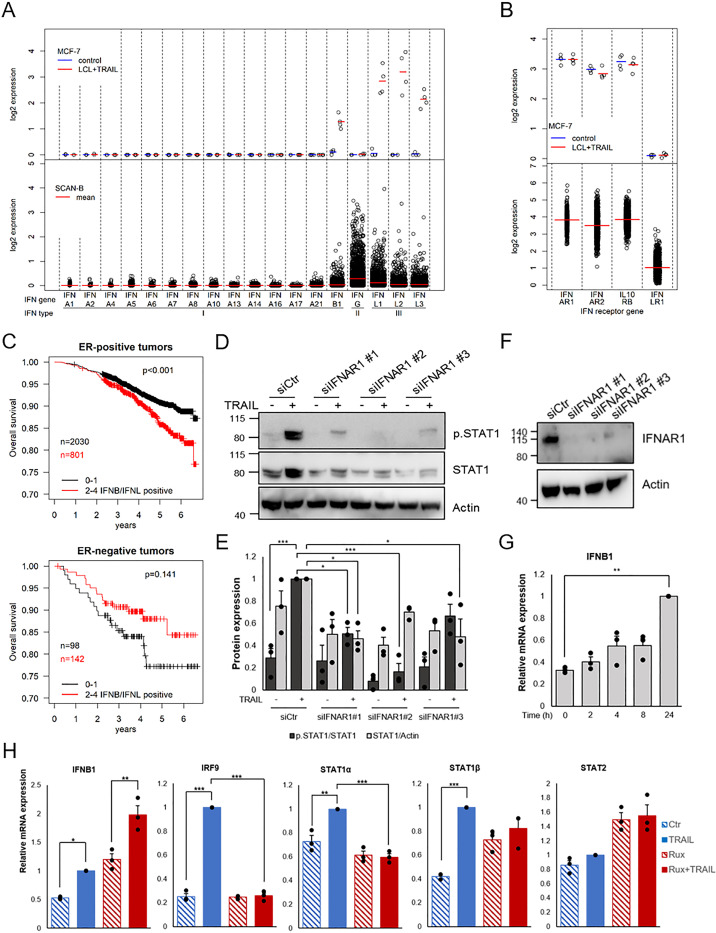
TRAIL-induced STAT1 phosphorylation is dependent on the IFN alpha/beta receptor. Expression of IFN genes (A) and IFN receptor genes (B) in MCF-7 cells treated with 10 μM LCL161 and 100 ng/mL TRAIL for 24 h and in tumors from the SCAN-B cohort as comparison. Each point represents log2 mRNA expression determined with RNA-seq of one sample/tumor and the mean is marked with a line. C) Kaplan-Meier curves of 2831 breast cancer patients with ER-positive tumors and 240 with ER-negative tumors from the SCAN-B cohort. The mRNA data for IFNB1 and type III IFNs (IFNL1-3) were retrieved and tumors were classified based on how many of these four genes had expression levels above baseline. Black line represent tumors with 0–1 and red line represent tumors with 2–4 IFN genes above baseline. The endpoint is overall survival. D) Knock-down of IFN alpha/beta receptor (IFNAR1) was carried out in MCF-7 cells using siRNA transfection prior to 24 h of treatment with TRAIL. Using Western blot, phosphorylated and total STAT1 protein levels were analyzed and knockdown is shown in F. E) Intensity of bands in D were quantified and normalized to TRAIL-treated cells that had been incubated with control siRNA. G and H) mRNA expression levels of IFN-related genes were analyzed from MCF-7 cells treated with TRAIL (100 ng/mL) for indicated time points (G) or pre-treated with Ruxolitinib (20 μM) and TRAIL for 24 h (H), using qRT-PCR. Data (mean ± SEM, three independent experiments) are log2 mRNA expression related to housekeeping genes and normalized to the 24 h sample for G, or to TRAIL treatment in the absence of Ruxolitinib for H. * denotes p<0.05, ** p<0.01, *** p<0.001 using ANOVA followed by Tukey’s honest significance test.

Janus kinase-mediated phosphorylation of STAT1 is a hallmark for IFN signaling via IFN receptors [26]. The RNA-seq data indicates that the type I IFNB1 and the type III IFNL1-3, but not the type II IFNG, genes are induced by LCL161 and TRAIL in MCF-7 cells (Fig 4A), suggesting that either type I or III IFN could be an autocrine mediator of the signaling. We therefore also looked at the expression of genes encoding the receptors for type I and type III IFNs (Fig 4B). Here we found that mRNAs encoding the type I receptors IFNAR1 and IFNAR2 as well as one of the type III receptors IL10RB were clearly detectable. However, mRNA encoding the other component of the type III receptor, IFNLR1, was not detected.

To get an indication if the IFNs seen after LCL161 and TRAIL treatment of MCF-7 cells are present in breast cancer tissue, RNA-seq data from the SCAN-B cohort of 3271 primary breast cancers diagnosed between 2010 and 2015 in Southern Sweden [36], were analyzed for IFN expression. As can be seen in Fig 4A the major IFN expressed in tumors is the type II IFNG, which is generally found in leukocytes and therefore conceivably reflects the amount of immune cells in the tumor. This IFN is not expressed in MCF-7 cells. The type III IFN genes induced in MCF-7 cells are all expressed in a number of breast cancers and of the type I IFNs, only IFNB1 was found in MCF-7 cells and it was also the most frequently expressed type I gene in breast cancers. The expression of IFNA genes is less pronounced with less than 10% of the tumors having detectable levels. The expression pattern for mRNAs encoding IFN receptors was similar in breast cancer and MCF-7 cells (Fig 4B).

Considering previous reports on the association of IFN with breast cancer prognosis we also analyzed the cohort for association with prognosis of the number of IFNB and IFNL genes, *i*.*e*. the IFN genes induced in MCF-7 cells, above detection level (Fig 4C). As can be seen for ER-positive breast cancer, the prognosis was worse if more than two of the genes could be detected compared to tumors where at most one IFNB or IFNL gene was expressed. The pattern was reversed in ER-negative cancers, but in this case the difference was not significant. The results are in line with other reports where IFN is associated with less favorable outcome in ER-positive and the opposite in ER-negative breast cancers [28–31].

Since mainly mRNA encoding type I receptors was detected in MCF-7 cells and IFNB1 was the type I IFN gene found to be upregulated upon LCL161 and TRAIL treatment, we hypothesized that induction of STAT1 phosphorylation may be mediated by IFNB1 autocrine signaling. To test this, MCF-7 cells were treated with siRNA targeting IFNAR1 which resulted in a suppression of LCL161 and TRAIL-mediated phosphorylation of STAT1 and increase in STAT1 protein levels (Fig 4D and 4E). We next examined the mRNA expression levels of IFNB1 after 0–24 hours of treatment with TRAIL (Fig 4G). TRAIL induced a gradual increase in IFNB1 expression. We also tested the effect of Ruxolitinib on TRAIL-mediated expression of IFN-regulated genes (Fig 4H). IFNB1 mRNA was increased by TRAIL also in the presence of Ruxolitinib. However, induction of IRF9 and STAT1α was suppressed. This would further support our hypothesis that TRAIL induces IFNB1 expression, which is followed by autocrine IFN stimulation and STAT1-mediated gene expression.

### Caspase-8, independently of its apoptotic activity, is critical for TRAIL-mediated IFN signaling

**Fig 5:**
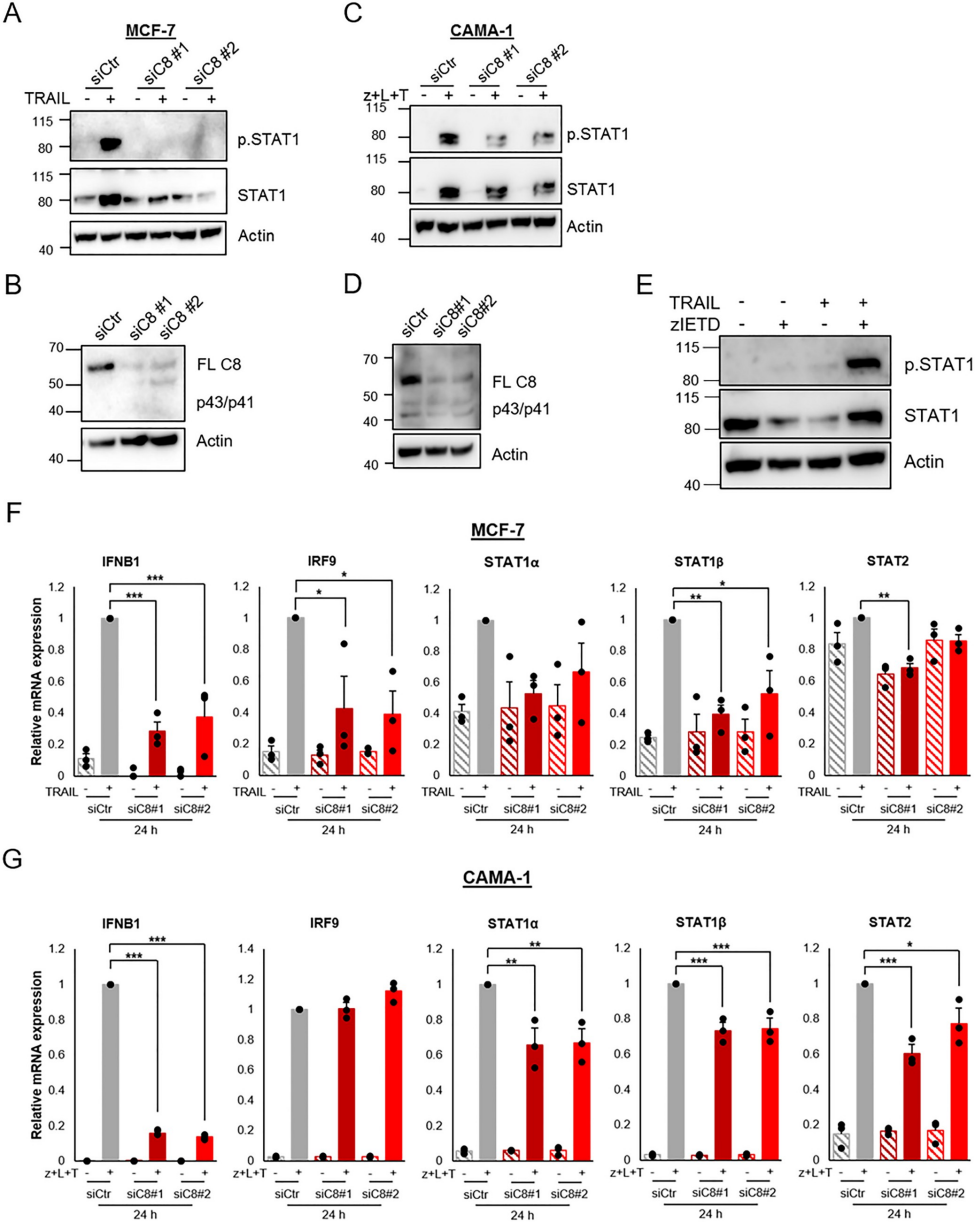
TRAIL induction of the IFN pathway in breast cancer cells is dependent on caspase-8. A) MCF-7 breast cancer cells were transfected with siRNA targeting caspase-8 followed by treatment with TRAIL (100 ng/mL) for 48 h. Total cell lysates were analyzed with Western blot for phosphorylated and total STAT1. B) Downregulation of caspase-8 with siRNA transfection of MCF-7 cells was validated. C) Total cell lysates from CAMA-1 cells transfected with siRNA targeting caspase-8 were treated for 48 h with LCL161 (10 μM), TRAIL (100 ng/mL), and zVAD-FMK (20 μM) were analyzed for phosphorylated and total STAT1 protein levels using Western blot. D) Downregulation of caspase-8 with siRNA was validated. E) MCF-7 cells were pre-treated with 20 μM zIETD-FMK for 5 min before 100 ng/mL TRAIL was added for 24 h. Using immunoblot, phosphorylated and total STAT1 protein levels were assessed. F and G) Caspase-8 was downregulated using siRNA in MCF-7 cells, which was followed by treatment with TRAIL (100 ng/mL) for 24 h (F), and in CAMA-1 cells followed by treatment with zVAD-FMK (20 μM), LCL161 (10 μM), and TRAIL (100 ng/mL) for 24 h (G). mRNA levels of indicated genes were thereafter analyzed with qRT-PCR. Data are mean ± SEM from three independent experiments, of log2 expression of indicated genes related to the expression of housekeeping genes and normalized to stimulation in cells treated with a control siRNA. * denotes p<0.05, ** p<0.01, *** p<0.001 using ANOVA followed by Tukey’s honest significance test.

Caspase inhibition did not suppress the induction of the IFN pathway. However, it has been described that caspase-8 has a scaffolding role, independent of its apoptotic activity, which is required for inflammatory signaling induced by TRAIL [22]. Hence, we examined the role of caspase-8 in TRAIL-induced IFN signaling in breast cancer cells. Downregulation of caspase-8 in MCF-7 cells completely abolished TRAIL-induced STAT1 phosphorylation and the increase in total STAT1 protein levels (Fig 5A and 5B). For CAMA-1, downregulation of caspase-8 decreased the phosphorylation of STAT1, whereas the effect on total STAT1 protein levels was not as convincing as in MCF-7 cells (Fig 5C and 5D). Since zVAD-FMK, used in Fig 1, is a broad caspase inhibitor we tested zIETD-FMK, which is more specific for caspase-8. As for zVAD-FMK this inhibitor had no suppressive effect on TRAIL-mediated STAT1 phosphorylation (Fig 5E), further underscoring that the effect of caspase-8 is independent of its apoptotic activity.

We also studied the role of caspase-8 for TRAIL-mediated expression of IFN-regulated genes. TRAIL treatment causes increases in IFNB1, IRF9, STAT1α and STAT1β mRNA expression (Fig 5F). Downregulation of caspase-8 in MCF-7 cells significantly decreased the TRAIL-stimulated increase in mRNA levels of IFNB1 and STAT1β (Fig 5F) and there were tendencies of the same effect for STAT1α and IRF9. In CAMA-1 cells, treatment for 24 hours with the combination of LCL161, TRAIL and zVAD-FMK resulted in elevated mRNA expression of all IFN-related genes investigated. As in MCF-7 cells, downregulation of caspase-8 significantly reduced the mRNA expression of IFNB1, STAT1α, STAT1β, and STAT2. However, IRF9 mRNA levels were unaffected (Fig 5G).

**Fig 6:**
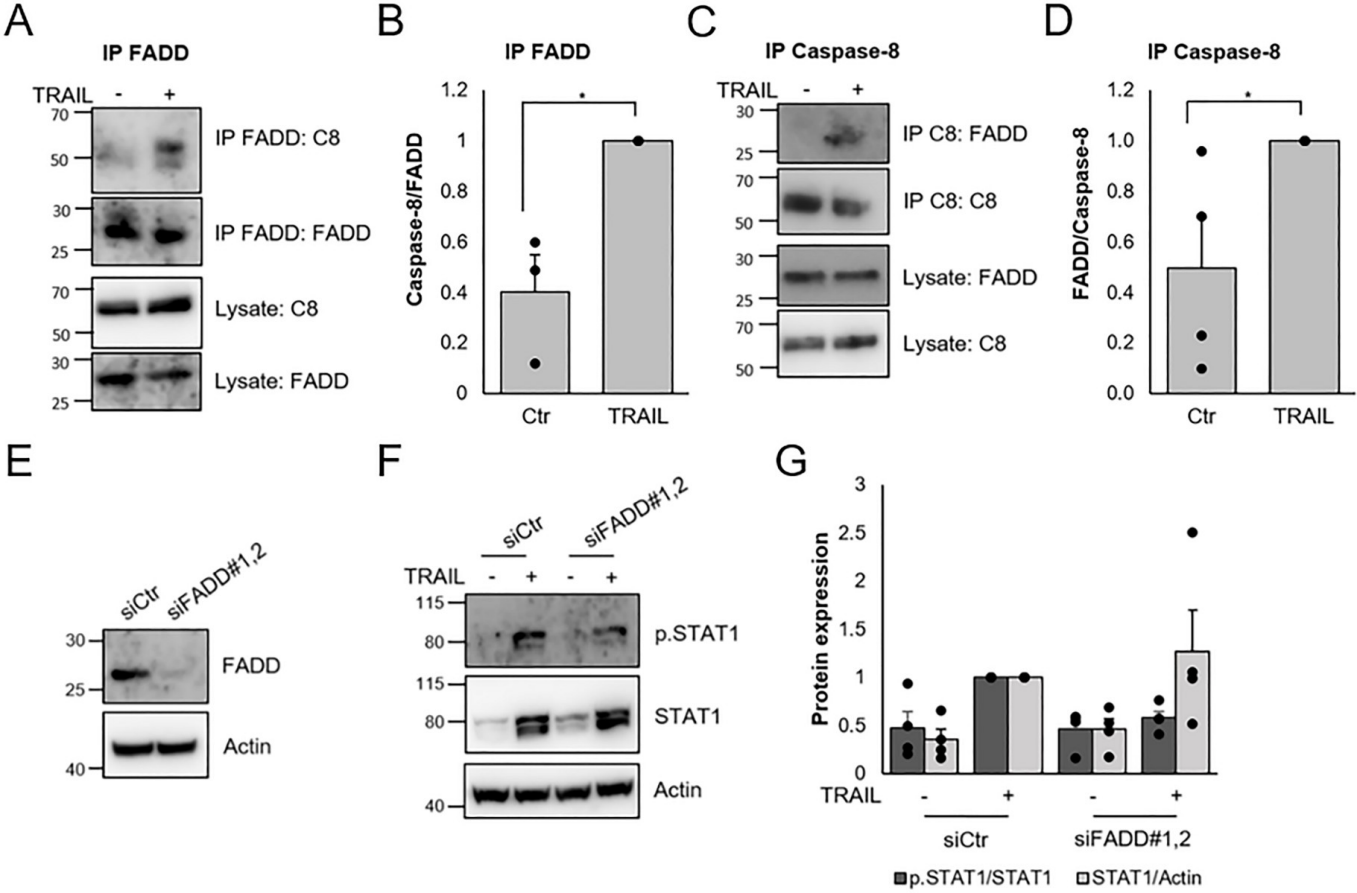
Involvement of FADD in TRAIL-mediated IFN signaling. A-D) MCF-7 cells were stimulated with 100 ng/ml TRAIL for 2 h in the presence of 20 μM zVAD-FMK and lysates were immunoprecipitated with antibodies towards FADD (A, B) or caspase-8 (C, D). Lysates and immunoprecipitates were subjected to Western blot for FADD and caspase-8 (A and C). Intensities in bands for the immunoprecipitates were quantified (B and D). E-G) MCF-7 cells were transfected for 72 h with a combination of two siRNAs targeting FADD (E) followed by treatment with TRAIL (100 ng/ml) for 48h. Lysates were subjected to Western blot with indicated antibodies (F) and intensities in bands were quantified (G). Data (B,D,G) are mean ± SEM with individual data points indicated and experiments were performed three (B) or four (D,G) times. * denotes p<0.05, using Student’s t-test.

Caspase-8 is considered to form a complex with FADD upon stimulation of death receptors. We found that FADD and caspase-8 could be co-immunoprecipitated in MCF-7 cells after stimulation for two hours with TRAIL (Fig 6A–6D). We also took the approach to downregulate FADD with siRNAs. In order to achieve substantial knockdown we had to incubate with two separate siRNAs simultaneously and prolong the siRNA incubation to 72 hours (Fig 6E). This resulted in a tendency to reduction of STAT1 phosphorylation seen after 48 hours of TRAIL stimulation (Fig 6F). However, the increase in total STAT1 was not affected and the magnitude of the effect was not as large as that obtained by caspase-8 knockdown.

### Neither RIP1 nor c-FLIP are critical for TRAIL-mediated STAT1 phosphorylation

**Fig 7:**
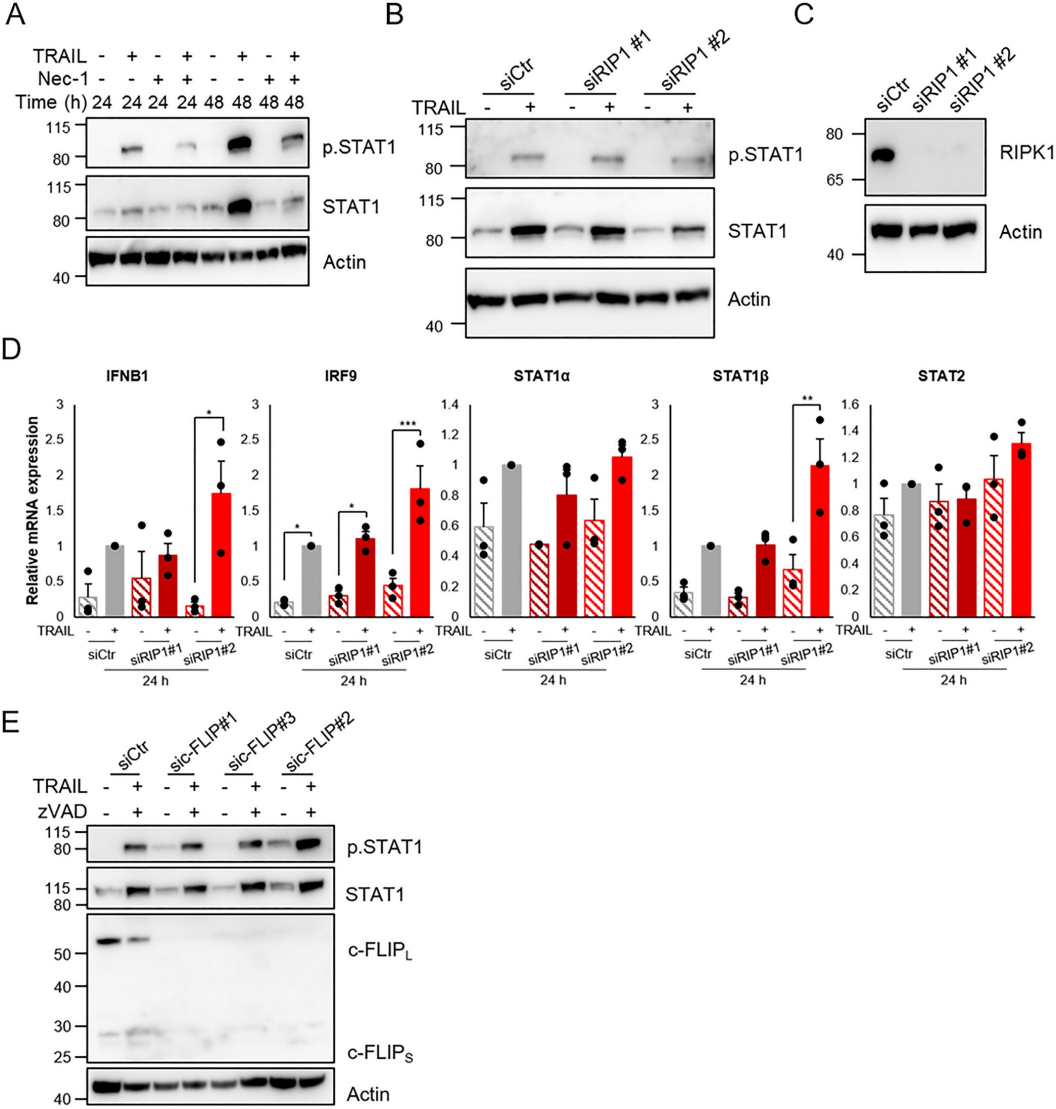
Involvement of RIP1 in TRAIL-mediated IFN signaling. A) MCF-7 cells were pre-treated with 10 μM Necrostatin-1 (Nec-1) before 100 ng/mL TRAIL was added for indicated time points. Protein levels of phosphorylated and total STAT1 were analyzed by immunoblot. B) RIP1 was downregulated using siRNAs and was followed by 24 h of TRAIL (100 ng/mL) treatment and Western blot to analyze protein levels of total STAT1 and phosphorylated STAT1. C) Downregulation of RIP1 was validated. D) After downregulation of c-FLIP using siRNAs, MCF-7 cells were pre-treated with zVAD-FMK (20 μM) and TRAIL (100 ng/mL) for 24 h. Total cell lysates were then used to analyze protein levels of phosphorylated and total STAT1 and c-FLIP splice variants (c-FLIP_L_ and c-FLIP_S_). * denotes p<0.05, ** p<0.01, *** p<0.001 using ANOVA followed by Tukey’s honest significance test.

We next investigated RIP1 kinase which has been described to mediate IFN-β production in macrophages [37], induce NF-κB-mediated IFN signaling in conjugation with FADD [38], and to be critical for TRAIL-mediated induction of cytokines [22, 39]. Inhibition of RIP1 kinase activity with necrostatin-1 (Nec-1) showed some tendency to suppression of the TRAIL effect on STAT1 (Fig 7A). However, downregulation of RIP1 with siRNA did not have an apparent effect on TRAIL-mediated STAT1 phosphorylation (Fig 7B and 7C) or induction of IFN-responsive genes (Fig 7D).

c-FLIP is a caspase-8-interacting protein that comes in splice variants that can either block or promote caspase-8 activity [40]. Given the role of caspase-8 we therefore tested whether downregulation of c-FLIP would influence the TRAIL-mediated activation of the IFN pathway. To exclude contributions of caspase-8 activity we included the pan-caspase inhibitor zVAD-FMK. The siRNAs downregulated both the short and long c-FLIP splice variants but had no effect on STAT1 phosphorylation induced by TRAIL (Fig 7E).

## Discussion

Here we show that treatment of CAMA-1 and MCF-7, two ER-positive and luminal-like breast cancer cell lines, with Smac mimetic LCL161 and TRAIL induces IFN signaling with phosphorylation of STAT1. The combination of Smac mimetics and death receptor stimulation elicits apoptosis in many cell types. For CAMA-1 cells it was necessary to block apoptosis by caspase inhibition for STAT1 phosphorylation to be detectable. Nevertheless, the effect was dependent on caspase-8 in both cell types since downregulation of this protein blocked STAT1 phosphorylation, but neither a pan-caspase inhibitor nor a more caspase-8 specific inhibitor suppressed the effect. Contrasting our results, it was previously shown that treatment of MCF-7 cells with the caspase-8 specific inhibitor zIETD-FMK reduced STAT1 phosphorylation following CD95 stimulation [41], suggesting different roles of caspase-8 in mediating induction of IFN signaling depending on which type of death receptor that is stimulated.

The data suggest that TRAIL induction of IFN signaling involves a non-apoptotic role of caspase-8. Such a role of caspase-8 has been shown to be critical for stimulation of cytokine production by TRAIL [21, 22] and CD95 [42] and it was suggested that caspase-8 in this setting is important as scaffold. Caspase-8 was needed for formation of the FADDosome, a complex involving FADD and RIP1, which mediated the induction of cytokines. Such a tentative scaffolding role of caspase-8 has also been demonstrated for DISC formation with recruitment of caspase-10 in HeLa cells after CD95 stimulation [43], and in regulation of the inflammasome by TLR3 signaling [44]. Moreover, the scaffold role of caspase-8 has been indicated *in vivo*, where mice bearing *Casp8*^*-/-*^*Mlkl*^*-/-*^ mutations were viable, whereas mice with catalytically inactive caspase-8 and *Mlkl*^*-/-*^ displayed embryonic lethality, which suggests a pro-apoptotic scaffolding function of inactive caspase-8 [45].

Our data highlight induction of IFN expression as an additional role for caspase-8, independent of its apoptotic activity, in death receptor-mediated signal transduction. In line with other reports we could detect an association of caspase-8 with FADD, but downregulation of FADD did not reduce induction of IFN signaling as efficiently as did knockdown of caspase-8. This may be related to less efficient downregulation of FADD, that low amounts of FADD are sufficient for the signal transduction to take place, or that there are other compensatory mechanisms mediating the caspase-8-dependent effect. Contrasting previous findings on TRAIL-mediated cytokine induction [21, 22] the effects of TRAIL on IFNB1 could take place in the absence of RIP1 and it was not blocked by Smac mimetics indicating that there are differences in the transduction pathways leading to the events.

**Fig 8:**
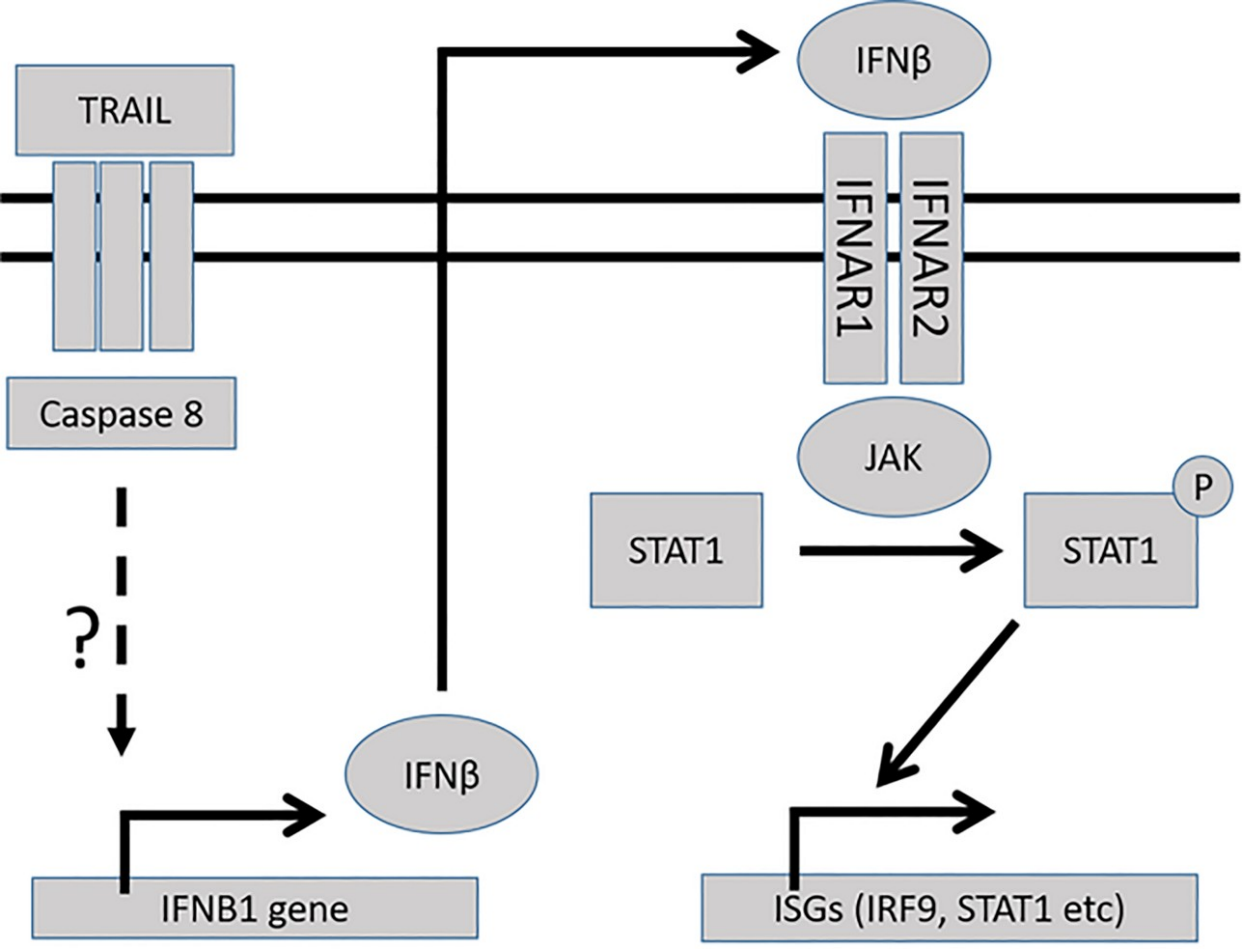
Hypothesis of the signaling pathway. The hypothesis suggests a pathway following TRAIL stimulation leading to induction of interferon-stimulated genes (ISGs).

For MCF-7 cells the phosphorylation of STAT1 was a rather late event, being detectable after 24 hours of stimulation. Furthermore, it was suppressed if the type I IFN receptor IFNAR1 was downregulated. This led us to postulate a hypothesis that there is an initial TRAIL-mediated IFNB1 induction followed by autocrine IFN stimulation, phosphorylation of STAT1, and activation of several IFN target genes, including IFNB1 itself (Fig 8). Autocrine IFN-β signaling has previously been described, for example in the monocytic leukemic cell line THP-1 after LPS stimulation [46], in dendritic cells after TLR activation [47], in glioma cells [48], and in human foreskin fibroblasts stimulated with TNF [49]. The hypothesis is further supported by our findings that TRAIL stimulation led to increases in IFNB1 mRNA levels even when Janus kinases were inhibited by Ruxolitinib, whereas the induction of other target genes, such as IRF9, was blocked by the inhibitor.

We do not yet know, besides caspase-8, what mediates TRAIL-induced IFNB1 expression. The IFNB1 gene can be regulated by several different transcription factors, including NF-κB, ATF-2, and c-Jun [50, 51]. NF-κB may be involved, at least in CAMA-1 cells, where Smac mimetics were necessary for the effect and LCL161 activated non-canonical NF-κB. However, in MCF-7 cells, TRAIL alone was sufficient for the effect and did not activate the non-canonical pathway. IRF3 and IRF7, which are well-established activators of IFNB1 transcription [35, 52], are other candidates. These proteins can be regulated by phosphorylation by the IKK-related kinases inhibitor of nuclear factor kappa-B kinase subunit epsilon (IKKε) and TANK-binding kinase 1 (TBK1) [53], which can be activated by several different mechanisms. For instance, LPS stimulation of TLR4 can activate IKKε/TBK1 via TRAF family member-associated NF-κB activator (TANK) [54] and dsRNA can do it via mitochondrial antiviral-signaling protein (MAVS) [55]. IKKε and TBK1 have also been described in TNF signaling, where ubiquitination of RIP1 enables recruitment of NEMO, IKKε, and TBK1 via TANK and nucleosome assembly protein (NAP1) [56]. It is possible that TRAIL signaling may involve similar mechanisms with a putative role for caspase-8.

Since TRAIL stimulation is one potential strategy to block breast cancer growth [57, 58], induction of IFN-β signaling in ER-positive MCF-7 breast cancer cells could be of importance. Several studies suggest that the expression of ISGs and activation of IFN signaling are correlated with poor outcome of ER-positive breast cancer. It is associated with treatment-induced resistance to radiotherapy and tamoxifen [28], increased progression of ER-positive AI-resistant breast cancer cells [29], poorer prognosis in ER-positive luminal B breast cancer [59], and we saw here that IFN-expression was associated with poorer survival in the SCAN-B cohort. In the light of these studies, TRAIL-mediated induction of IFN-β and subsequent autocrine IFN-β signaling may be of significance for treatment strategies for ER-positive breast cancer.

## Materials and methods

### Cell culture and reagents

MCF-7 and CAMA-1 breast cancer cells (from American Type Culture Collection) were cultured in cell culture dishes (Falcon) with RPMI-1640 medium (Corning) supplemented with fetal bovine serum (FBS) (10%, Biosera), sodium pyruvate (1 mM, Corning), penicillin (100 IU/mL, Corning), and streptomycin (100 μg/mL, Corning). The cell line identities were confirmed with a Multiplex Human Cell Line Authentication Test (Multiplexion) and tests for Mycoplasma infection were done every other month (Eurofins). When indicated, cells were treated with LCL161 (MedChem Express) and/or TRAIL (Millipore) where 0.1% DMSO (Sigma-Aldrich) or ddH_2_O was used as control. In addition, pre-treatment with inhibitors against caspases (zVAD-FMK, Enzo Life Sciences), caspase-8 (zIETD-FMK, MedChem Express), RIP1 kinase (Necrostatin-1, Sigma-Aldrich), and Janus kinases (Ruxolitinib, Selleckchem) was carried out when indicated.

### Cell viability analysis

For WST-1 analysis, 6,000 MCF-7 cells were seeded in 100 μL medium per well in 96-well culture plates. The following day, the cells were treated for 48 or 72 h by adding 100 μL medium containing indicated compounds. WST-1 (20 μL, Roche) was added when 4 h of the treatment remained. Cell viability was analyzed by a WST-1 assay as described by the manufacturer’s protocol using a Synergy 2 Microplate Reader and Gen5 Reader Control and Data Analysis software (BioTek).

### Western blot

CAMA-1 or MCF-7 cells were seeded in 60 mm cell culture dishes, at a density of 750,000 cells. The subsequent day indicated compounds were added. After treatment, cells were washed in ice-cold PBS before being lysed using RIPA buffer (160 mM NaCl, 1% Triton X-100, 1% sodium-deoxycholate, 0.1% SDS, 1 mM EGTA, 10 mM TRIS-HCl (pH 7.2), and 1 mM EDTA) containing EDTA-free Complete protease inhibitor (40 μg/mL, Roche). Cell lysates were centrifuged at 14,000 *x g*, 4°C, for 10 min and supernatants were mixed with 2X Sample buffer containing 10% DTT. An equal amount of protein from each sample was added to NuPAGE 10% Bis-Tris Plus gels (Invitrogen) and was separated by electrophoresis. The proteins were subsequently transferred to Immobilon-P polyvinylidene difluoride membranes (Millipore) which then were blocked through incubation in 5% milk-powder in PBS. This was followed by incubation with primary antibodies against phosphorylated STAT1 (1:400, #9167), total STAT1 (1:400, #9172), FADD (1:400, #27825), caspase-8 (1:300, #9746), and FLIP (1:400, #56343) all from Cell Signaling Technology; p100/p52 (1:300, ab131539) and IFNAR1 (1:400, ab124764) from Abcam; cIAP1 (1:200, AF8181, R&D Systems); actin (1:2,000, #0869100, MP Biomedicals); and RIP1 (1:500, #610458, BD Biosciences). Afterwards, the membranes were incubated with horseradish peroxidase-labeled secondary antibody (1:5,000) against goat (P0449, Dako), mouse or rabbit IgG (NAS931V and NA934V, respectively, both from GE Healthcare Life Sciences). Using Luminata Forte Western HRP Substrate (Millipore), according to the manufacturer’s instructions, the chemiluminescence was detected using either Amersham Imager 600 (GE Healthcare Life Sciences) or a LAS-1,000 camera and Image Reader LAS-1,000 Pro Software (both from Fujifilm). Quantifications were performed using ImageJ.

### Co-immunoprecipitation

For co-immunoprecipitation, 2.5 x 10^6^ cells were seeded in 10 cm cell culture dishes. After 48 h, the medium was changed and the cells were pre-incubated with 20 μM zVAD-FMK for 5 min before addition of indicated compounds. Cells were collected and lysed as described for Western Blot. Cell lysates were incubated with caspase-8 antibodies (1:75, #9746, Cell Signaling) or FADD antibodies (1:75, MA120168, Thermo Scientific) overnight at 4°C followed by using μMACS Protein G Microbeads and MACS Separation Columns (Miltenyi Biotec).

### Transfection of siRNA

CAMA-1 or MCF-7 breast cancer cells (750,000 cells per 60 mm plate) were seeded in medium without antibiotics. The cells were incubated in OptiMEM (Gibco) containing 2 μL/mL Lipofectamine-2,000 (Invitrogen) and 40 nM of Stealth siRNAs against either NIK (HSS113309 (#1), HSS113310 (#2), HSS189765 (#3)), IFNAR1 (HSS105226 (#1), HSS105227 (#2), HSS105228 (#3)), RIP1 (HSS112846 (#1), HSS112847 (#2)), or FLIP (HSS189605 (#1), HSS189607 (#2), HSS189606 (#3)) all from Invitrogen, or 5 nM Silencer Select siRNAs against caspase-8 (s2426 (#1), s2427 (#2)) or a combination of siRNAs against FADD (s16705 and s16706) from Thermo Fisher, for 30, 48 or 72 h.

### Microarray and RNA-sequencing analyses

Two million MCF-7 cells were treated with LCL161 and TRAIL. Cells were harvested after 24 h of treatment. Subsequently, RNA was extracted using the RNeasy Mini Kit (Qiagen). The RNA-Sequencing was performed at the Center for Translational Genomics, SciLifeLab Facility, Lund University. Briefly, libraries were constructed with the TruSeq® Stranded mRNA Library Prep (Illumina) and sequencing was performed on a NextSeq 500 (Illumina). Alignments and sequence counting were done with a HISAT2-StringTie pipeline using hg19 as reference genome. The data have been deposited at GEO (GSE122979). Data analyses were performed with R.

For RNA-seq data the log2 of the FPKM data after addition of 1 was used. Only sequences with a RefSeq prescript NM was used for the analyses. For gene set enrichment analysis the Hallmarks collection from the Molecular Signature Database (MSigDb, version 6.1 http://www.gseamsigdb.org/gsea/msigdb/index.jsp) was used [32].

### Analysis with qPCR

Using the RNeasy kit (Qiagen) RNA was extracted from cells that had been transfected and treated as indicated. This was followed by DNase treatment (RQ1 RNase-free DNase, Promega) according to the manufacturer’s instructions. 2 μg RNA per sample was used for cDNA synthesis, using either MultiScribe Reverse Transcriptase (Applied Biosystems) for experiments where the effect of siCaspase-8 was examined, or SuperScript III Reverse Transcriptase (Thermo Fisher) for other qPCR analyses. For amplification of cDNA by qPCR in QuantStudio 7 Flex Real-Time PCR System (Thermo Fisher), the SYBR Green PCR Master Mix (Applied Biosystems) was used. Following primers were used: IFNB1, IRF9, STAT1α, STAT1β, and STAT2 (forward IFNB1: 5’-TTGCTCTCCTGTTGTGCTTC-3’, reverse IFNB1: 5’-TCAAAGTTCATCCTGTCCTTG-3’, forward IRF9: 5’-GAGCCACAGGAAGTTACAGACA-3’, reverse IRF9: 5’-ATGAAGGTGAGCAGCAGTGAG-3’, forward STAT1α: 5’-CCAATGGAACTTGATGGCCC -3’ reverse STAT1α: 5’-CAGAGCCCACTATCCGAGAC-3’, forward STAT1β: 5’-TGATGGCCCTAAAGGAACTG -3’, reverse STAT1β: 5’-AGGCTGGCTTGAGGTTTGTA-3’, forward STAT2: 5’-CTCCATTTCTTTCTTCCATTCC-3’, reverse STAT2: 5’-CTTCCTATCCATCCCTTTCTTC-3’), all from Invitrogen Life Sciences, and were designed using the Primer3 software. For normalization of gene expression, primers against SDHA, YWHAZ, and UBC mRNAs were used (forward SDHA: 5’-TGGGAACAAGAGGGCATCTG-3’, reverse SDHA: 5’-CCACCACTGCATCAAATTCATG-3’, forward YWHAZ: 5’-ACTTTTGGTACATTGTGGCTTCAA-3’, reverse YWHAZ: 5’-CCGCCAGGACAAACCAGTAT-3’, forward UBC: 5’-ATTTGGGTCGCGGTTCTT-3’, reverse UBC: 5’-TGCCTTGACATTCTCGATGGT-3’), all from Invitrogen Life Sciences. The relative gene expression was quantified by using the comparative C_T_ method [60], using three replicates of each sample.

### Statistical analysis

Statistical analyses, except for global mRNA data, were performed using IBM SPSS Statistics 26 where significance of difference was tested by Student’s t-test (two groups) or one-way ANOVA, followed by Tukey’s HSD test (multiple groups). Differences were considered significant if the p-value is below 0.05. Statistical and computational analyses of global mRNA data were performed with R 3.6.1. Differences in gene expression were analyzed with t-test. Analysis of gene set enrichment was done with Fisher’s test.

### Public data sets

Data from the SCAN-B cohort of 3271 primary breast cancers diagnosed between 2010 and 2015 in Southern Sweden [36] were retrieved from GEO (GSE96058).

## Supporting information

S1 Raw images Original, uncropped images of Western blots used in the figures. (PDF)

S1 Fig Extended heat map of gene sets after LCL161+TRAIL treatment in MCF-7 cells. This enlarged version of the heat map in Fig 1C, illustrating the expression of genes in three Hallmarks (MSigDb) gene sets, also contains explanatory gene names. The gene sets included are “TNFA SIGNALING VIA NFKB”, “INTERFERON ALPHA RESPONSE”, and “INTERFERON GAMMA RESPONSE”.
(TIF)
