# Supplementary material for: Induction of interferon-β and interferon signaling by TRAIL and Smac mimetics via caspase-8 in breast cancer cells
Source: PLoS One. 2021 Mar 26;16(3):e0248175. doi: 10.1371/journal.pone.0248175 (PMC7996988; doi:10.1371/journal.pone.0248175)

Supplementary: Figure 1

1D

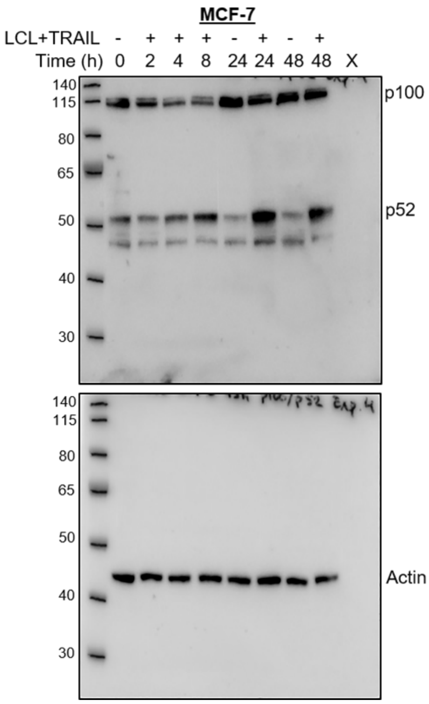

1F

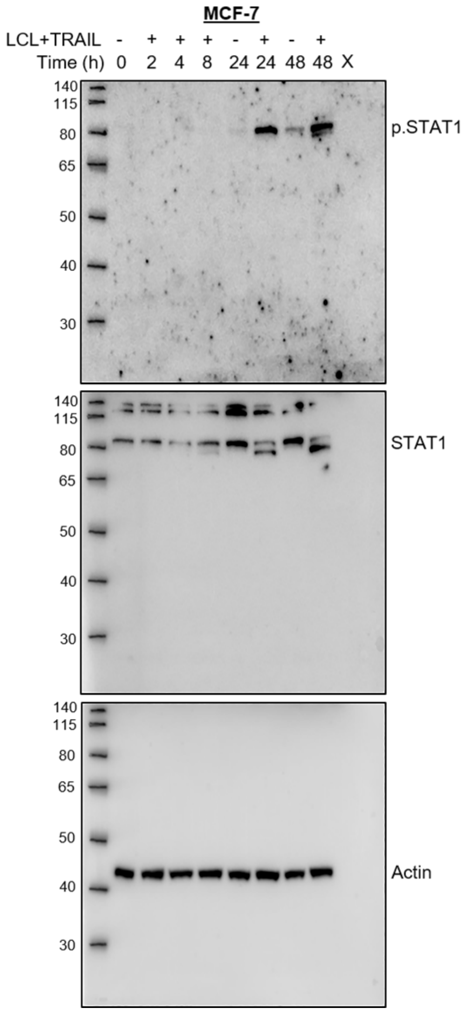

1G

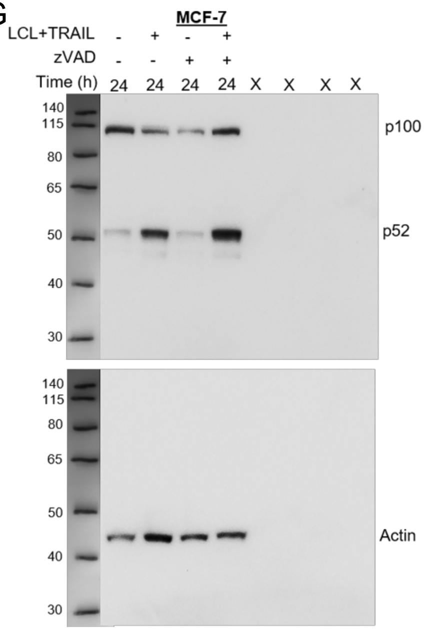

1H

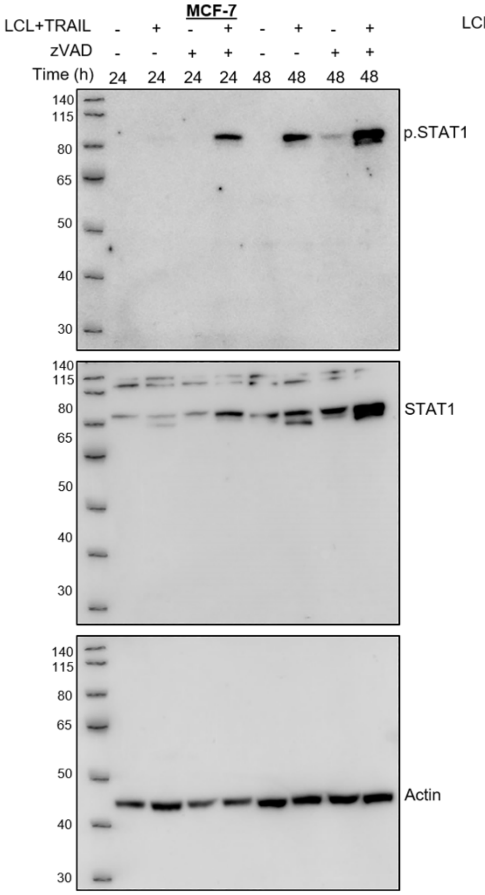

1J

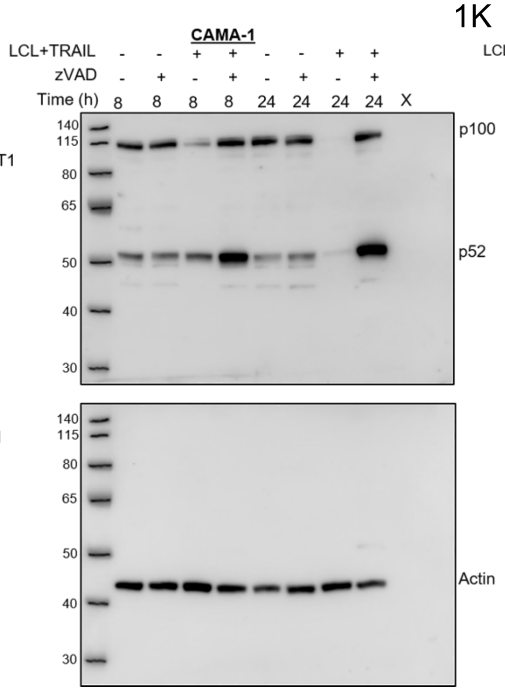

1K

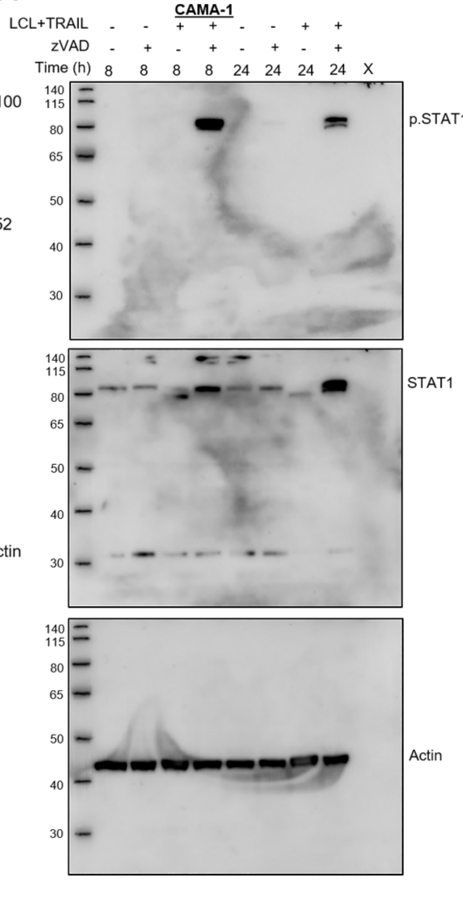

Supplementary: Figure 2

2A 2B 2C

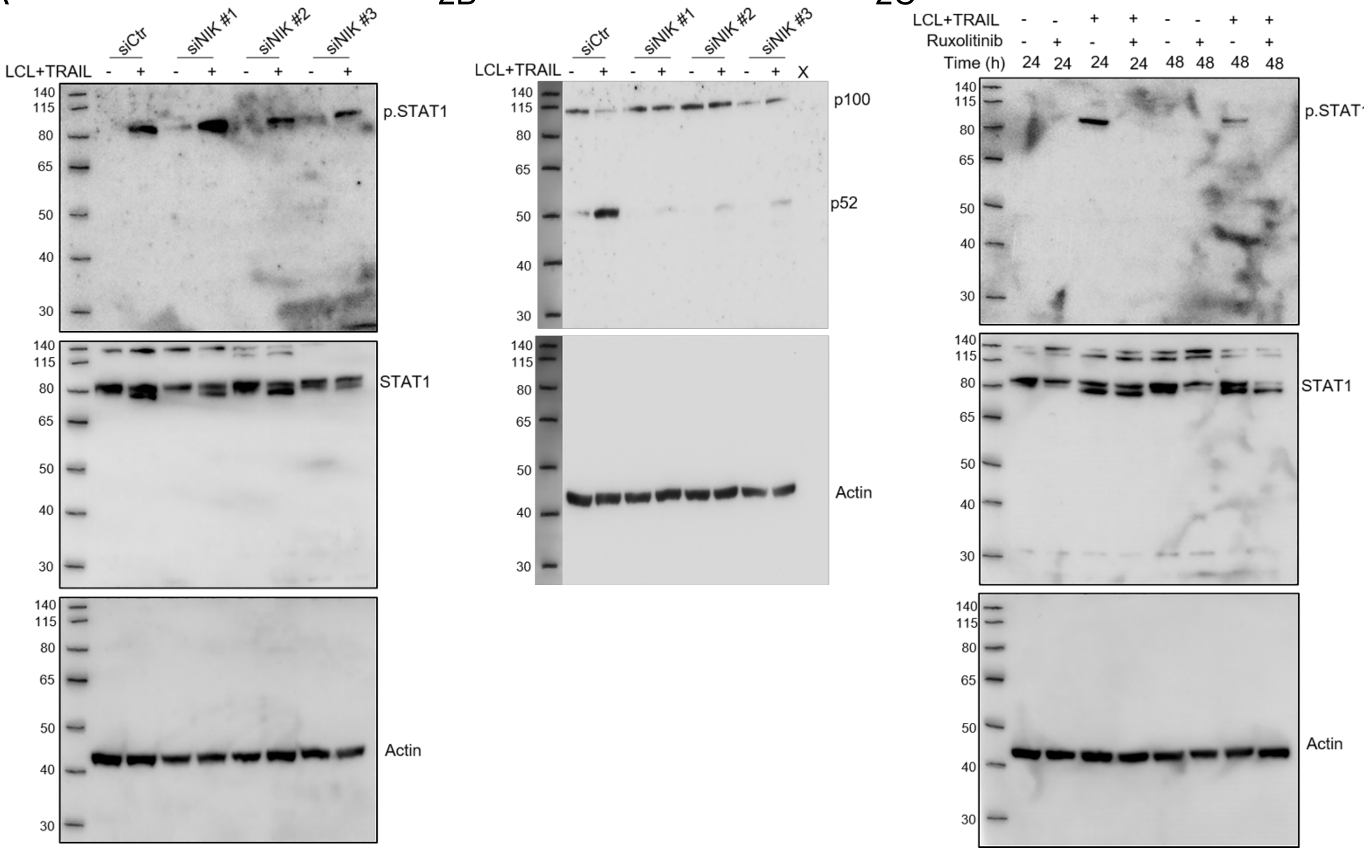

Supplementary: Figure 3

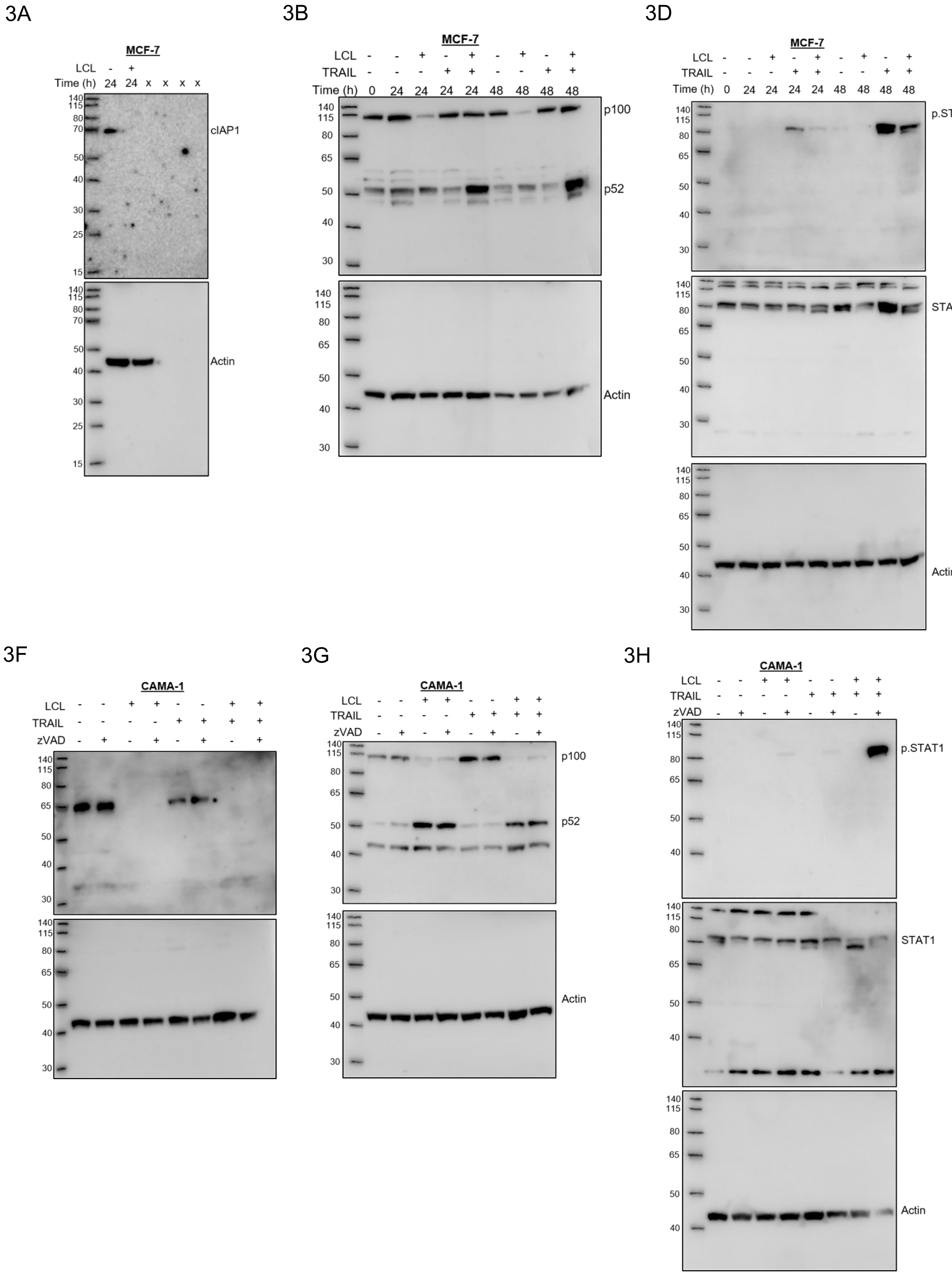

Supplementary: Figure 4

4D

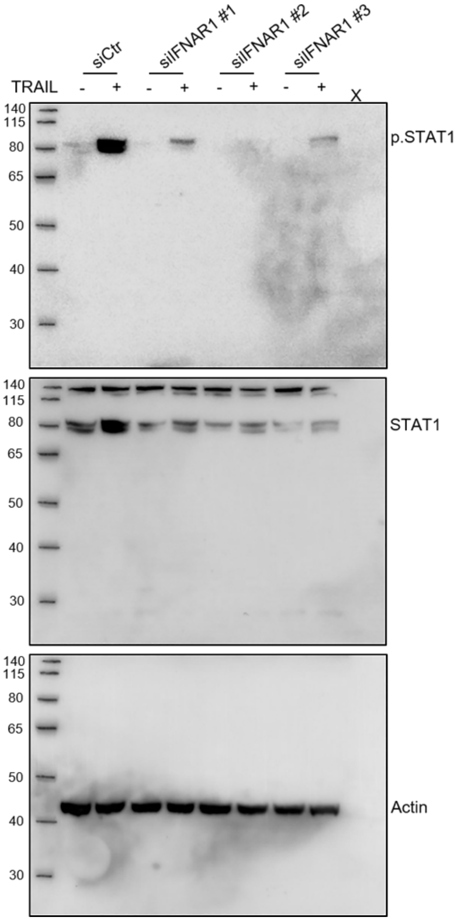

4F

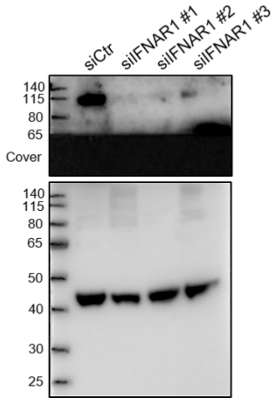

Supplementary: Figure 5

5A

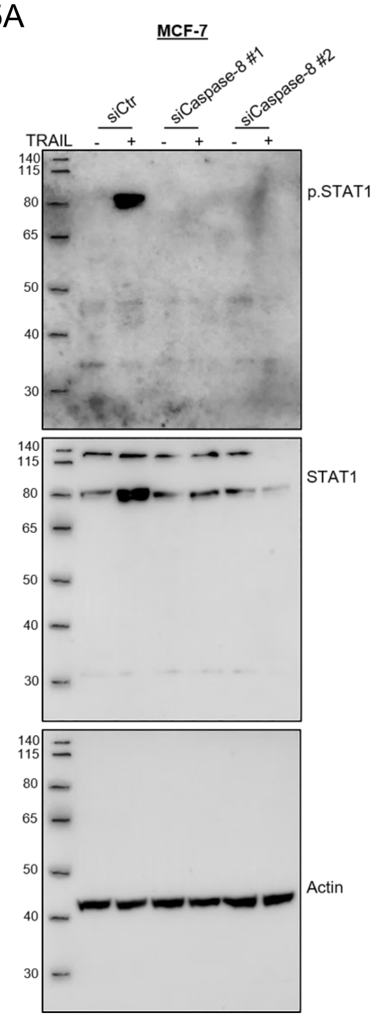

5B

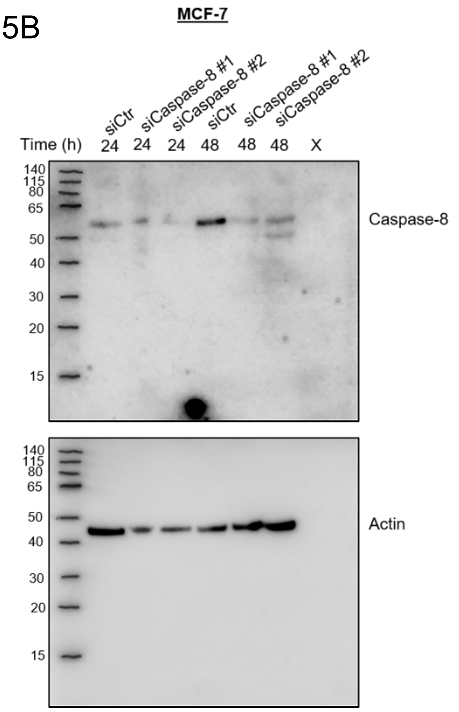

5C

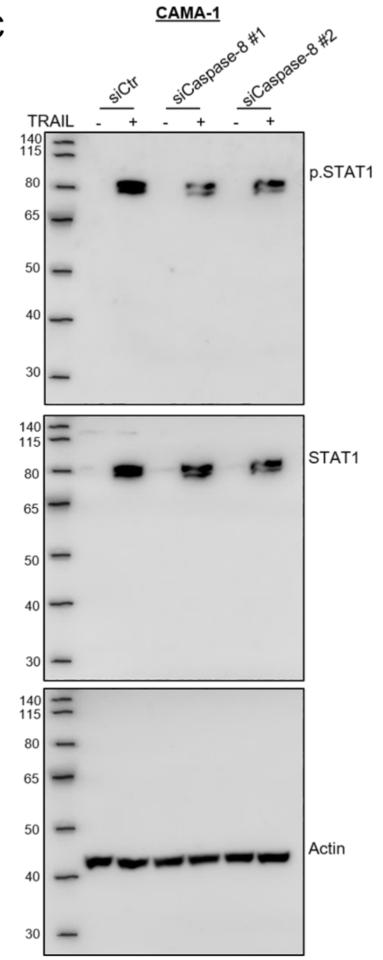

5D

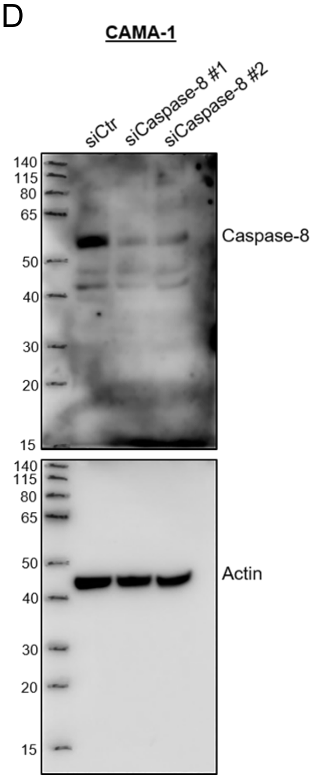

5E

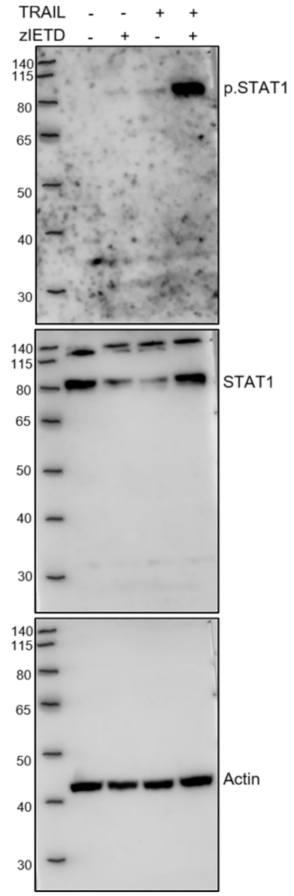

Supplementary: Figure 6

6A

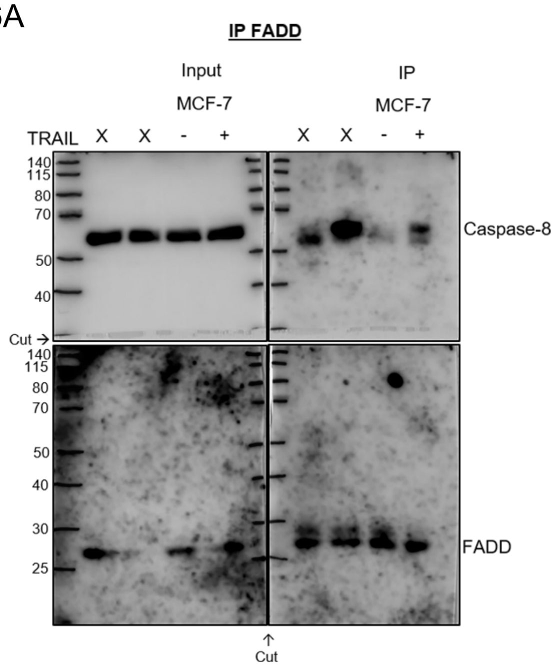

6C

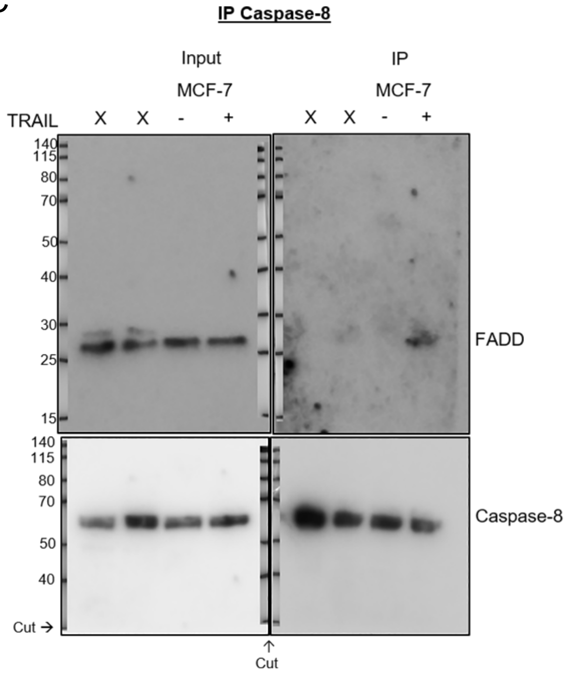

6E

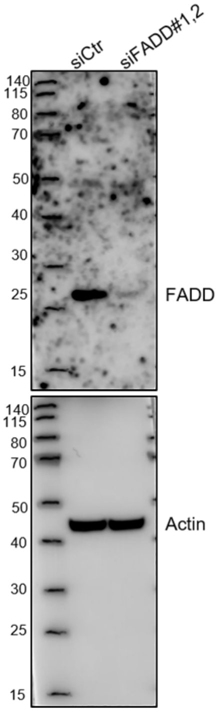

6F

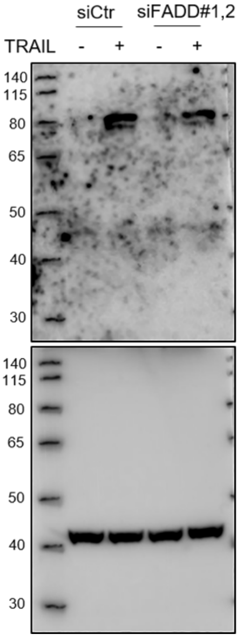

Supplementary: Figure 7

7A

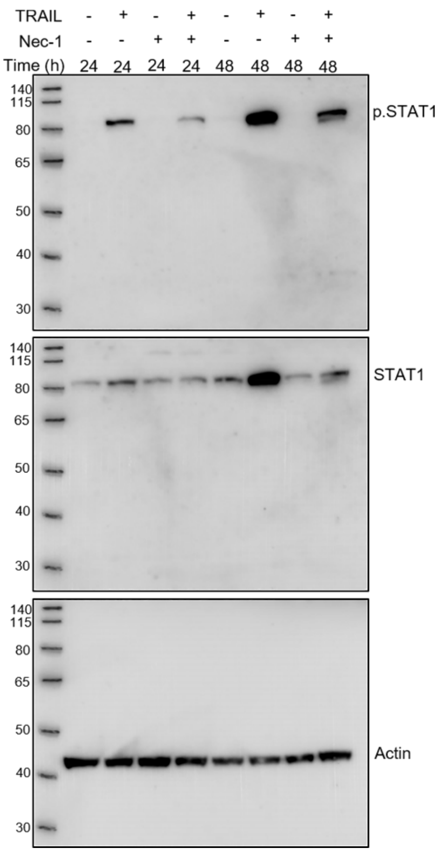

7B

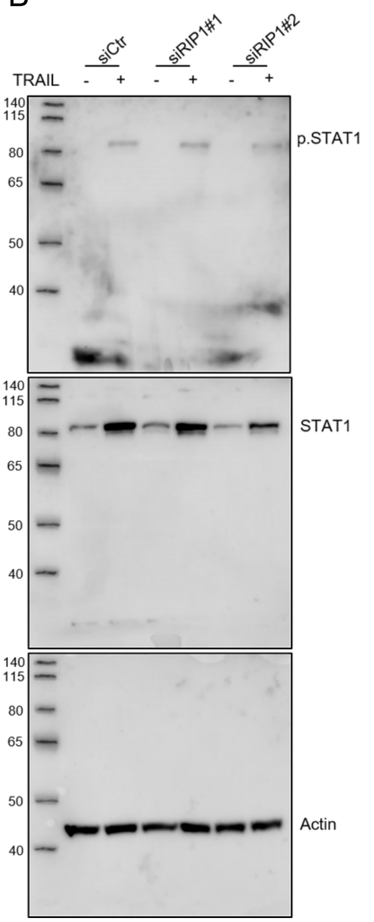

7E

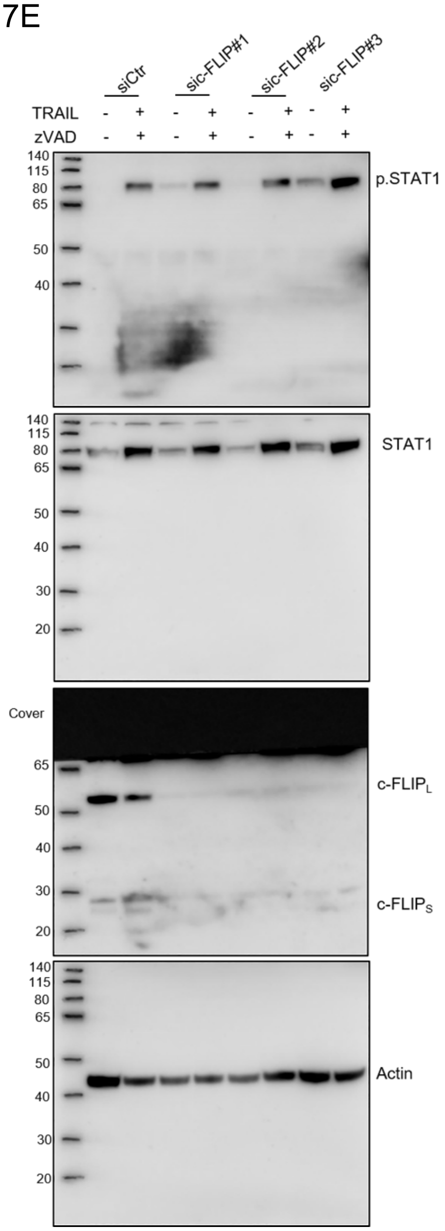

7C

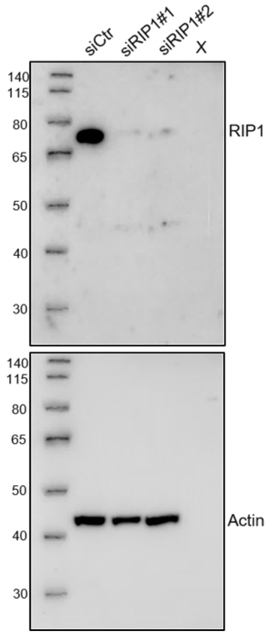

Supplement: S1 Raw images — (PDF) [file pone.0248175.s001.pdf]
