# Supplementary figures and images for: Induction of interferon-β and interferon signaling by TRAIL and Smac mimetics via caspase-8 in breast cancer cells
Source: PLoS One. 2021 Mar 26;16(3):e0248175. doi: 10.1371/journal.pone.0248175 (PMC7996988; doi:10.1371/journal.pone.0248175)

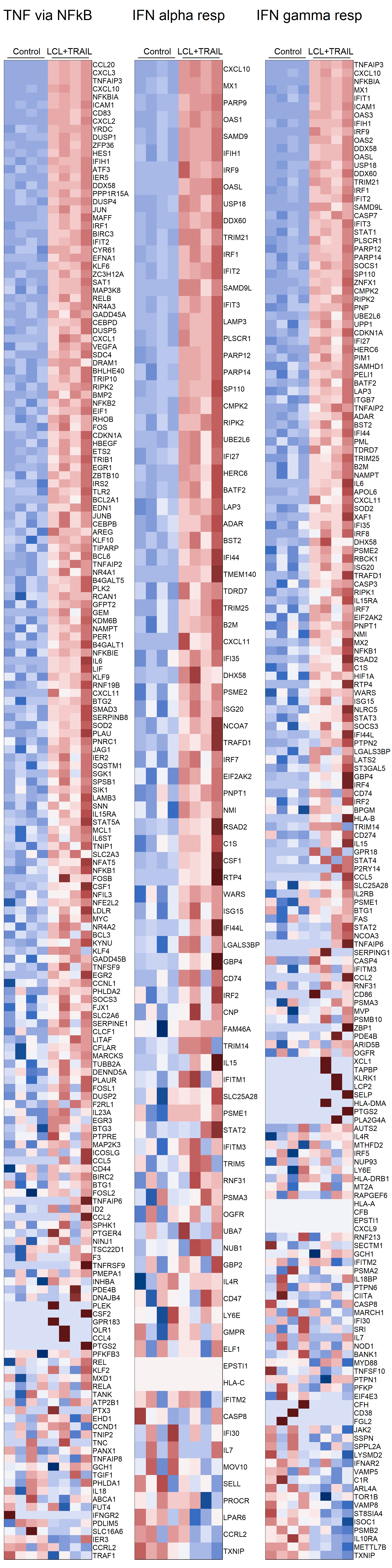

Supplement: S1 Fig — This enlarged version of the heat map in Fig 1C, illustrating the expression of genes in three Hallmarks (MSigDb) gene sets, also contains explanatory gene names. The gene sets included are “TNFA SIGNALING VIA NFKB”, “INTERFERON ALPHA RESPONSE”, and “INTERFERON GAMMA RESPONSE”. (TIF) [file pone.0248175.s002.tif]
